# A holistic prognostic model for leukemia-free survival after allogeneic transplantation in acute leukemia

**DOI:** 10.1038/s41409-026-02950-w

**Published:** 2026-07-02

**Authors:** Alexandros Spyridonidis, Allain Thibeault Ferhat, Jacques Emmanuel Galimard, Thomas Schroeder, Régis Peffault de Latour, Nicolaus Kröger, Didier Blaise, Matthias Stelljes, Matthias Eder, Tobias Gedde-Dahl, Igor Wolfgang Blau, Ibrahim Yakoub-Agha, Edouard Forcade, Anne Huynh, Francesca Kinsella, Hélène Labussière-Wallet, Jakob Passweg, Robert Zeiser, Mohamed Houhou, Bipin Savani, Roni Shouval, Jurjen Versluis, Mohamad Mohty, Fabio Ciceri, Annalisa Ruggeri

**Affiliations:** 1 University of Patras, Patras, Greece; 2 EBMT Paris Study Unit, Hôpital Saint Antoine, Paris, France; 3 3University Hospital Essen, Essen, Germany; 4 Saint-Louis Hospital, BMT Unit, Paris, France; 5 University Hospital Eppendorf, Hamburg, Germany; 6 Transplant and cellular immunotherapy program, Department of Hematology, Institut Paoli Calmettes, Aix Marseille Univ, Marseille, France; 7 Department of Medicine A, University Hospital Münster Germany, Münster, Germany; 8 Hannover Medical School, Hannover, Germany; 9 Oslo University Hospital, Rikshospitalet, Oslo, Norway; 10 Medizinische Klinik m. S. Hämatologie, Onkologie und Tumorimmunologie, Berlin, Germany; 11 CHU de Lille, Lille, France; 12 CHU Bordeaux, Hopital Haut-Leveque, Pessac, France; 13 CHU - Institut Universitaire du Cancer Toulouse, Toulouse, France; 14 Birmingham Centre for Cellular Therapy and Transplant (BCCTT), Stoke, United Kingdom; 15 Service d’ Hématologie Hôpital Lyon Sud, Oullins-Pierre-Bénite, France; 16 University Hospital Basel, Basel, Switzerland; 17 University of Freiburg, Freiburg, Germany; 18 Vanderbilt University Medical Center, Nashville, TN USA; 19 Department of Medicine, Memorial Sloan Kettering Cancer Center, Weill Cornell Medicine, New York, NY USA; 20 Erasmus MC, Rotterdam, Netherlands; 21 Sorbonne University, Saint-Antoine Hospital, Paris, France; 22 IRCCS Ospedale San Raffaele s.r.l., Milano, Italy

**Keywords:** Risk factors, Medical research

## Abstract

Allogeneic hematopoietic cell transplantation (allo-HCT) can cure acute leukemia, but relapse and non-relapse mortality restrict its success. Existing models focus on single prognostic areas and are not designed to predict leukemia-free survival (LFS), the outcome that best reflects allo-HCT’s curative intent. In this large retrospective cohort of allografted AL patients (*N* = 24,317), we combined key pre-transplant patient, disease, and treatment factors into a practical holistic H-score to predict post–allo-HCT LFS. Component weights were derived from Cox-regression hazard ratios in a training cohort (*N* = 19,029). The model was validated in a geographically split testing cohort (N = 4760), with outcomes assessed using Kaplan-Meier analysis and multivariate Cox models. In the overall cohort (*N* = 24,317), 2-year overall survival and LFS were 64% and 56%, respectively. The H-score stratified patients into four risk groups, with 2-year LFS ranging from 66.2% in the low-risk group to 32.0% in the very high-risk group. The H-score was the strongest independent predictor of LFS (*p* < 0.0001), outperforming individual indices and offering more refined risk stratification. The H-score was also an independent predictor of overall survival, relapse, and non-relapse mortality. While individual prediction is limited, the H-score aids patient counseling and provides a useful baseline for comparing new transplant treatments.

## Introduction

Although allogeneic hematopoietic cell transplantation (allo-HCT) is a potentially curative approach for acute leukemia, its potential for cure is offset by the risks of non-relapse mortality (NRM) and disease relapse (REL). Outcomes are influenced by a complex interplay of factors, including patient-related variables (e.g., age, performance status, comorbidities), disease characteristics (e.g., risk profile, remission status at transplantation), donor-related factors (e.g., human leukocyte antigen matching), physician-driven choices (e.g., conditioning regimen and intensity, cell source), and less quantifiable elements such as frailty and social vulnerability [1]. Several scoring systems have been developed to standardize the assessment of the complex, multi-dimensional factors related to the patient, disease, and transplant procedure, such as the HCT-specific Comorbidity Index (HCT-CI), the Disease Risk Index (DRI), and the Transplant Conditioning Intensity (TCI) score [2–4]. Indeed, these scoring systems have been validated as independent predictors of specific post-transplant outcomes [5–7]. However, each model is confined to a specific prognostic domain, overlooks other relevant variables, and provides limited capacity for personalized post-transplant survival prediction. Pragmatically, these scores complement clinical judgment and serve as valuable tools for therapeutic decision-making, contributing significantly to advancements in allo-HCT. Nevertheless, physicians must counsel patients with the most accurate and realistic information regarding their chances of cure. This clinical need formed the rationale for the present study. Leveraging a large, contemporary dataset from the European Society for Blood and Marrow Transplantation (EBMT) registry, we evaluated whether readily available pre-transplant scores encompassing patient-, disease-, and treatment-specific factors could be used synergistically to predict post-transplant outcomes, with LFS designated as the primary endpoint.

## Methods

### Study design, population and outcomes

We conducted a retrospective analysis using data from the EBMT Registry, a collaborative network of over 500 routinely audited transplantation centers that annually report all consecutive HCTs and follow-up data in a standardized manner, following patient authorization through informed consent. Inclusion criteria comprised adult patients ( ≥ 18 years) diagnosed with acute myeloid leukemia (AML) or acute lymphoblastic leukemia (ALL) who underwent their first allo-HCT between 2015 and 2021, using peripheral blood stem cells or bone marrow from any donor type (excluding cord blood), and with complete data available to calculate the HCT-CI, refined DRI (rDRI), and TCI scores based on published definitions. The primary objective was to predict LFS at 2 years. Secondary objectives included estimating overall survival (OS), NRM, and REL incidence. All outcomes were measured from the time of allo-HCT. OS was defined as the time to death from any cause, LFS as survival without leukemia progression or relapse, NRM as death without previous relapse/progression, and relapse as leukemia recurrence. Two-year probabilities of OS and LFS were calculated using the Kaplan-Meier estimate, while cumulative incidence functions were used to estimate NRM and REL, accounting for competing risks. Baseline patient characteristics were summarized descriptively.

### Model development and validation

To construct and internally validate a prognostic score for LFS, the dataset was divided into a training cohort (*n* = 19,029; 78%) and a testing cohort (*n* = 4760; 22%) based on geographical location, with the German cohort selected for testing as it represented the largest national dataset. This approach was consistent with the Transparent Reporting of a Multivariable Prediction Model for Individual Prognosis or Diagnosis (TRIPOD) recommendations [8]. Using the training dataset, we conducted a multivariate Cox proportional hazards regression to estimate hazard ratios (HRs) and 95% confidence intervals (CIs) for LFS, incorporating factors known to be prognostically relevant. The resulting HRs were used to assign weighted scores to age, Karnofsky Performance Status (KPS), HCT-CI, DRI, and TCI, with the sum of these weights forming the composite Holistic Score (H-score). Specifically, variables with an HR between 1.0 and 1.5 were assigned a weight of 1; those with an HR > 1.5 and ≤2.0 were assigned a weight of 2; and variables with an HR > 2.0 were assigned a weight of 3. The prognostic value of the H-score was assessed in both the training and testing datasets using Kaplan–Meier survival analysis and multivariate Cox proportional hazards modeling. The analysis was adjusted for additional transplant-related covariates, including donor type, stem cell source, female-to-male donor–recipient mismatch, cytomegalovirus (CMV) serostatus mismatch, and transplant center, the latter included as a frailty effect to account for center-specific variability. Internal validation of the model was conducted in the testing cohort using a bootstrap resampling procedure, in which the model was refitted on 1000 resamples to evaluate the stability and reliability of the prognostic estimates [9]. Harrell’s concordance index (C-index) and corresponding confidence intervals were calculated in the testing cohort for the H-score and each individual score. All statistical analyses were performed using SPSS version 24.0 (SPSS Inc., Chicago, IL, USA) and R statistical software version 4.2.3 (R Foundation for Statistical Computing, Vienna, Austria).

## Results

### Characteristics of the study population

**Fig. 1:**
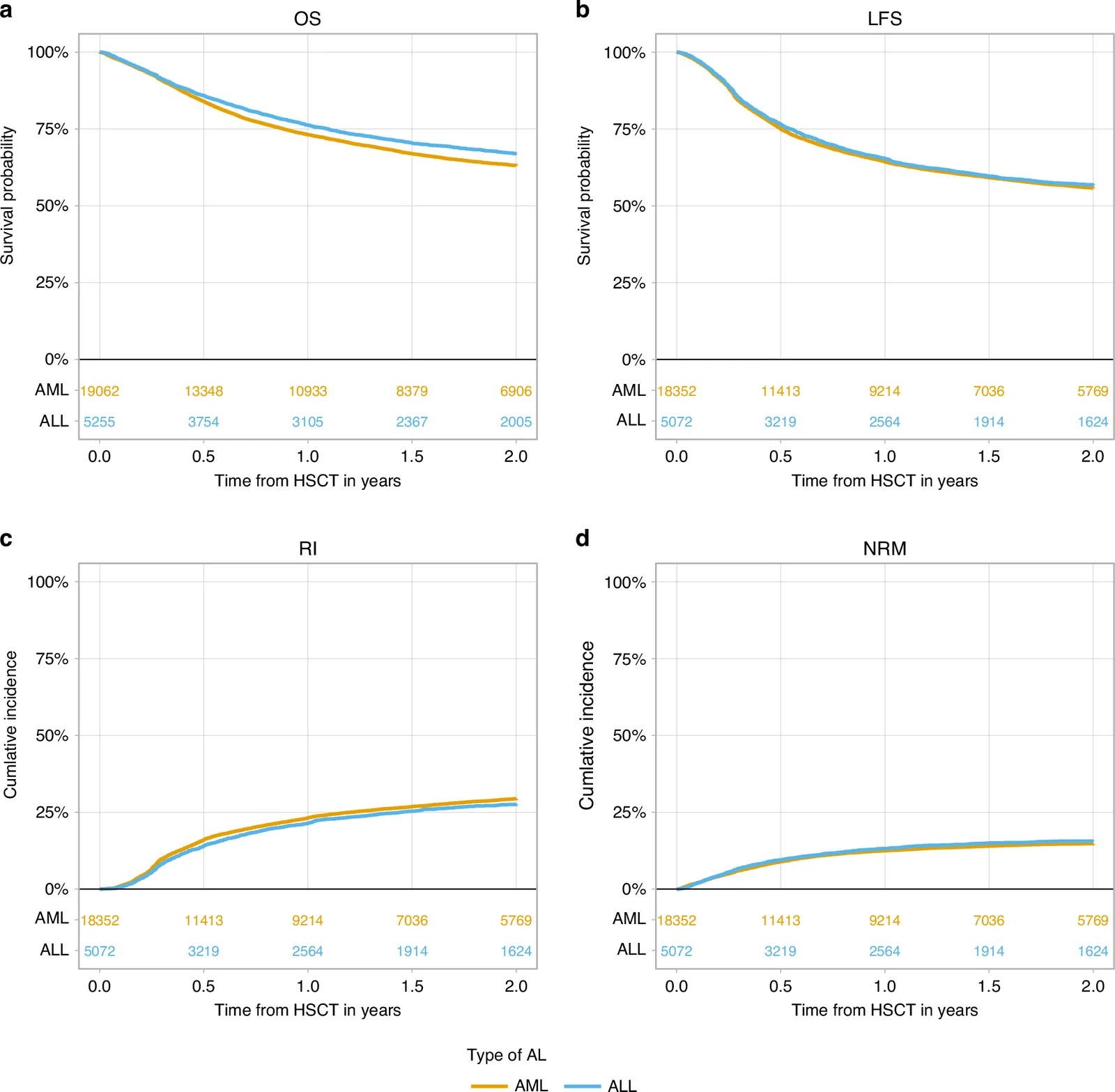
Transplant outcomes for the whole population. Kaplan-Meier curves showing overall survival (**a**) and leukemia-free survival (**b**) and cumulative incidence curves for relapse incidence (**c**) and non-relapse mortality (**d**) for all patients included in the study and stratified by leukemia type (acute myeloid leukemia [AML] vs acute lymphoblastic leukemia [ALL]).

Patient, disease, and transplant characteristics are summarized for the overall cohort (*n* = 24,317) and separately for patients with AML (*n* = 19,062) and ALL (*n* = 5255) in Table 1, with additional details provided in Supplementary Table S1. The median age of patients was 53.2 years (interquartile range: 39.9–62.3); 55.9% were male, of which 16.5% received grafts from female donors. A KPS below 90% was observed in 27% of patients, and 24% had multiple comorbidities with a HCT-CI score of ≥3. Most patients (71%) underwent transplantation in first complete remission (CR1), while 15.6% were in second or subsequent remission ( ≥ CR2), and 13.2% had active disease at the time of transplantation. According to the Disease Risk Index (DRI), 4.7% of patients were classified as low risk, 64% as intermediate risk, 27% as high risk, and 4.8% as very high risk. Peripheral blood was the stem cell source in 90.4% of cases, while bone marrow was used in 9.6%. Cytomegalovirus seronegativity in both donor and recipient was observed in 23% of transplants. Notable differences were observed between AML and ALL patients, with ALL patients being significantly younger (median age: 40.6 vs. 55.9 years; *p* < 0.001) and more likely to receive a total body irradiation–based conditioning regimen (Supplementary Table 1). The median follow-up was 2.4 years (95% CI: 2.3–2.4), with a median of 2.4 years for AML patients and 2.3 years for ALL patients. For the entire cohort, the estimated 2-year OS was 64% (95% CI: 63.3–64.6), while LFS was 56% (95% CI: 55.3–56.7). The 2-year cumulative incidence of REL was 29% (95% CI: 28.4–29.7), while NRM was 15% (95% CI: 14.5–15.5). Estimated outcomes by leukemia subtype are summarized in Supplementary Table S2, with corresponding survival curves shown in Fig. 1.

**Table 1:** Population characteristics.

Variable	Overall	AML	ALL
***N***	24,317	19,062	5255
**Age at HCT**			
years, median (IQR)	53.2 (39.9, 62.3)	55.9 (44.5, 63.7)	40.6 (28.0, 53.0)
**Age at HCT**			
<=55 years	13,227 (54%)	9092 (48%)	4135 (79%)
>55 years	11,090 (46%)	9970 (52%)	1120 (21%)
**KPS**			
>= 90	17,705 (73%)	13,807 (72%)	3898 (74%)
< 90	6612 (27%)	5255 (28%)	1357 (26%)
**HCT-CI**			
0	12,818 (53%)	9742 (51%)	3076 (59%)
1-2	5595 (23%)	4409 (23%)	1186 (23%)
>=3	5904 (24%)	4911 (26%)	993 (19%)
**TCI**			
4-6	6113 (25%)	3907 (20%)	2206 (42%)
2.5-3.5	11,558 (48%)	9139 (48%)	2419 (46%)
1-2	6646 (27%)	6016 (32%)	630 (12%)
**DRI**			
Low	1155 (4.7%)	1155 (6.1%)	0 (0%)
Intermediate	15,461 (64%)	11,648 (61%)	3813 (73%)
High	6538 (27%)	5346 (28%)	1192 (23%)
Very high	1163 (4.8%)	913 (4.8%)	250 (4.8%)
**Donor**			
MSD	7353 (30%)	5528 (29%)	1825 (35%)
Haplo	4459 (18%)	3521 (18%)	938 (18%)
UD 9/10	2506 (10%)	1962 (10%)	544 (10%)
UD 10/10	9999 (41%)	8051 (42%)	1948 (37%)
**Cell source**			
PB	21,976 (90.4%)	17,405 (91%)	4571 (87%)
BM	2341 (9.6%)	1657 (8.7%)	684 (13%)
**Female to male**			
No	20,074 (83%)	15,864 (83%)	4210 (80%)
Yes	4158 (17%)	3135 (17%)	1023 (20%)
Missing	85	63	22
**CMV donor/patient**			
Neg/ Neg	5497 (23%)	4228 (23%)	1269 (25%)
Other	18,365 (77%)	14,490 (77%)	3875 (75%)
Missing	455	344	111

*N* number of patients, *AML* acute myeloid leukemia, *ALL* acute lymphoblastic leukemia, *HCT* hematopoietic cell transplantation, *HCT-CI* hematopoietic cell transplantation–specific comorbidity index, *TCI* transplant conditioning intensity, *KPS* Karnofsky Performance Status, *MSD* matched sibling donor, *UD* unrelated donor, *9/10* 9-out-of-10 HLA-matched unrelated donor, *BM* bone marrow, *PB* peripheral blood, *CMV* cytomegalovirus.

### Development of the H-score

**Fig. 2:**
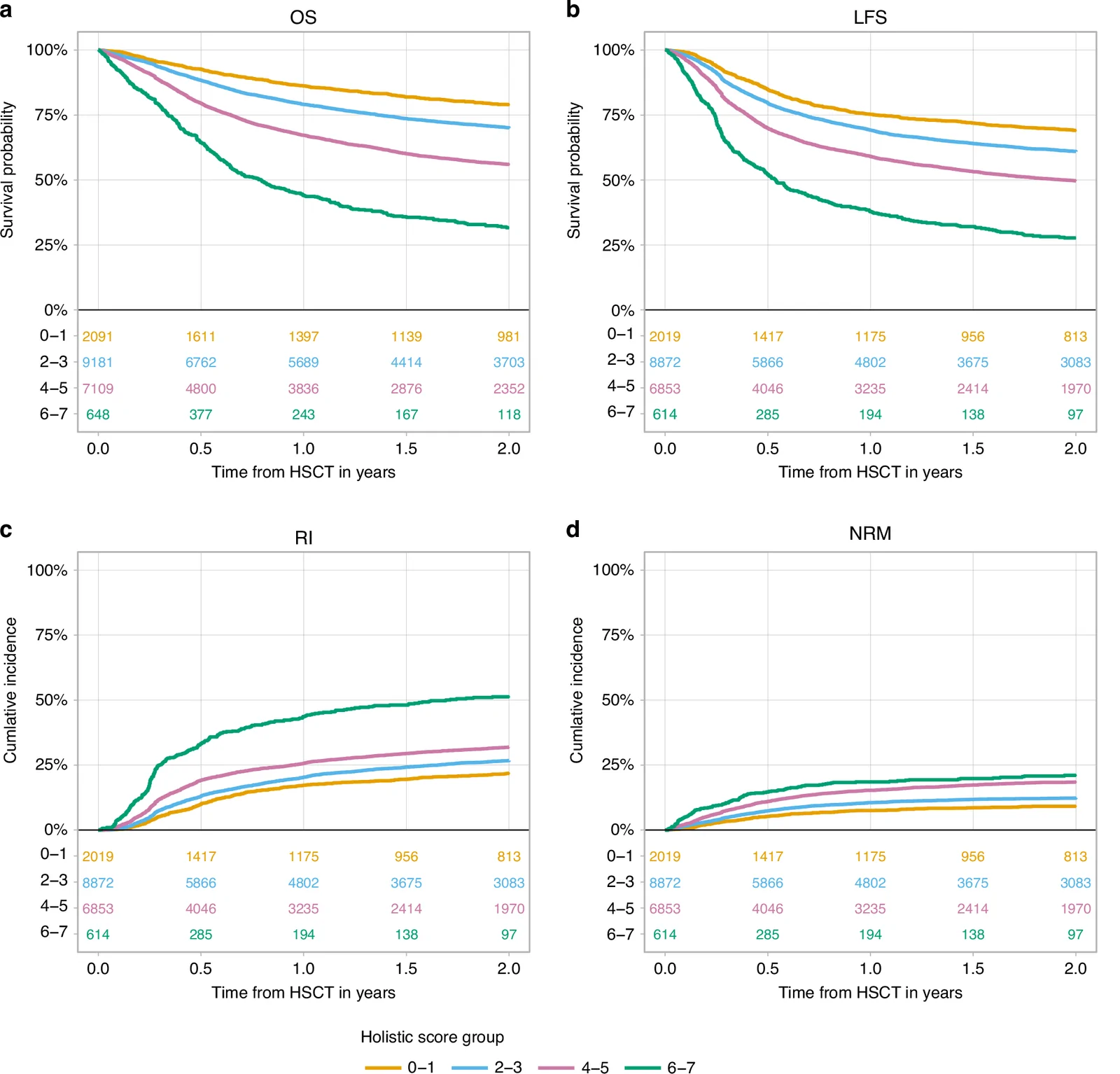
Transplant outcomes according to H-score in the training population. Kaplan-Meier curves showing overall survival (**a**) and leukemia-free survival (**b**) and cumulative incidence curves for relapse incidence (**c**) and non-relapse mortality (**d**) stratified by Holistic-score category (H-score) in the training dataset.

**Fig. 3:**
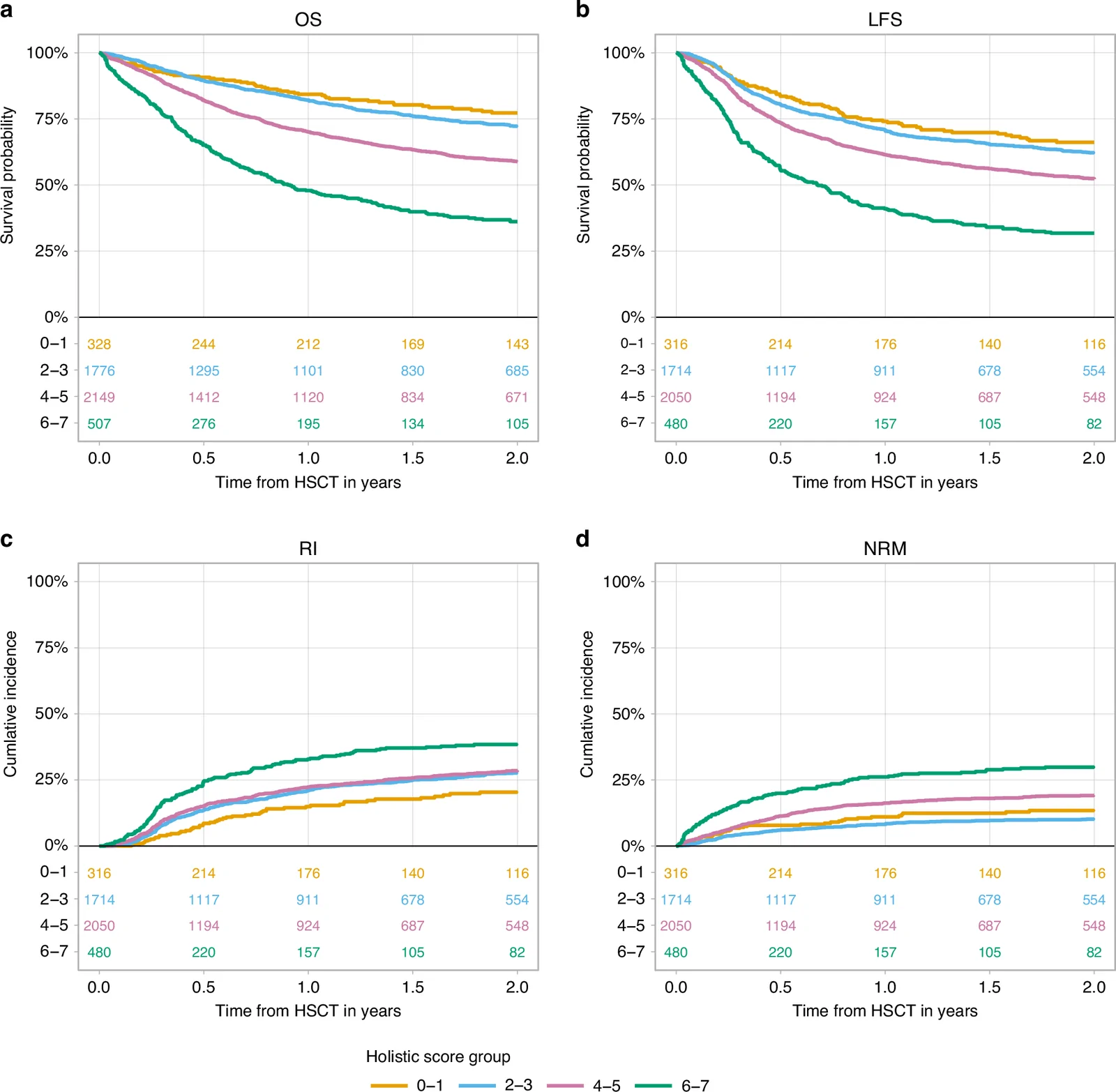
Transplant outcomes according to H-score in the testing population. Kaplan-Meier curves showing overall survival (**a**) and leukemia-free survival (**b**) and cumulative incidence curves for relapse incidence (**c**) and non-relapse mortality (**d**) stratified by Holistic-score category (H-score) in the testing dataset.

In unadjusted analyses, the HCT-CI, DRI, and TCI scores were each significantly associated with post-transplant outcomes, as shown in Fig. 2 (testing dataset), Fig. 3 (training dataset) and Supplementary Table S2. We then performed a multivariate Cox regression analysis for LFS using the training dataset (*n* = 19,029; 78% of the cohort), adjusting for HCT-CI, DRI, and TCI, along with additional known outcome-related factors not captured by these scores. (Supplementary Table S3). Age, KPS, HCT-CI, DRI, and TCI were identified as significant, independent predictors of LFS, highlighting their additive prognostic value (Table 2). To develop the new Holistic Score (H-score), we categorized the refined HRs for LFS from the training dataset as large (HR ≥ 2.0), intermediate (1.2 ≤ HR < 2.0), or small (1.0 ≤ HR < 1.2), and assigned corresponding integer weights of 3, 2, and 1, respectively. Accordingly, we assigned 0 points for age ≤55 years, Karnofsky score ≥90, HCT-CI of 0, low DRI, and TCI scores of 4–6; 1 point for age >55 years, HCT-CI ≥ 1, intermediate DRI, and TCI scores of 1–3.5; 2 points for Karnofsky score <90 and high DRI; and 3 points for very high DRI. The resulting H-score ranged from 0 to 7, with a median of 3 in the overall cohort, 4 in the AML group (range: 0–7), and 3 in the ALL group (range: 1–7). After consolidating score values that did not significantly differ in their association with LFS, four risk categories were defined: low (H-score 0–1), intermediate (2–3), high (4–5), and very high (6–7).

**Table 2:** Multivariate analysis on LFS in the training dataset and H-score value.

Variable		HR (95% CI)	*p*	H-score
**Age**	<=55			0
	>55	1.15 (1.09–1.22)	**<0.001**	1
**KPS**	>= 90			0
	<90	1.2 (1.14–1.27)	**<0.001**	1
**HCT-CI**	0			0
	1–2	1.07 (1.01–1.13)	**0.029**	1
	>=3	1.23 (1.16–1.3)	**<0.001**	1
**DRI**	Low			0
	Int	1.11 (0.98–1.26)	0.088	1
	High	1.96 (1.73–2.22)	**<0.001**	2
	Very high	3.74 (3.22–4.34)	**<0.001**	3
**TCI**	4–6			0
	2.5–3.5	1.04 (0.98–1.11)	0.197	1
	1–2	1.14 (1.06–1.23)	**0.001**	1

### Performance of the H-score for LFS

The H-score stratified patients into four risk groups, with 2-year LFS probabilities decreasing gradually from 69.1 to 27.7% in the training dataset and from 66.2 to 32.0% in the testing dataset, corresponding to the low- and very high-risk groups, respectively. (Supplementary Table S4) In a risk-adjusted Cox model that included the H-score alongside other established prognostic covariates, LFS declined in a systematic, statistically significant, and stepwise fashion across increasing H-score risk groups (Table 3). In this multivariate analysis, the H-score emerged as the strongest independent predictor of LFS in both training and testing datasets. In the testing cohort, however, separation between the low-risk (0–1) and intermediate-risk (2–3) categories was borderline (HR 1.24; 95% CI: 1.00–1.55; *p* = 0.054), likely reflecting the smaller validation sample, fewer events in the low-risk group, and differences in baseline patient and transplant characteristics in the geographically distinct validation cohort. Nevertheless, discrimination remained robust for the higher-risk categories. In the same multivariate model, the use of a mismatched 9/10 unrelated donor (UD) was associated with a modest yet statistically significant reduction in LFS (Table 3).

**Table 3:** Multivariate analysis of LFS including H-score in the training and testing dataset.

Variable	Level	Training HR (95% CI)	*P*	Testing HR (95% CI)	*P*
**H-score**	0–1				
	2–3	1.36 (1.25–1.49)	**<0.001**	1.24 (1.0–1.55)	0.054
	4–5	2.01 (1.84–2.2)	**<0.001**	1.91 (1.53–2.38)	**<0.001**
	6–7	3.87 (3.4–4.39)	**<0.001**	3.72 (2.92–4.75)	**<0.001**
**Donor type**	MSD				
	Haplo	1.02 (0.95–1.09)	0.545	1.02 (0.86–1.21)	0.85
	UD 10/10	0.98 (0.93–1.04)	0.566	0.98 (0.87–1.1)	0.753
	UD 9/10	1.1 (1.07–1.19)	**0.026**	1.2 (1.02–1.41)	**0.03**
**Cell source**	BM				
	PBSC	1.08(1.1–1.18)	**0.047**	0.76 (0.6–0.96)	**0.021**
**Female to male**	No				
	Yes	1.02 (0.96–1.08)	0.623	1.13 (0.99–1.29)	0.063
**CMV serostatus**	Neg to Neg				
	Other	1.07 (1.01–1.14)	**0.023**	1.01 (0.91–1.11)	0.886

We evaluated the H-score’s ability to predict LFS by calculating the concordance statistic (c-statistic) in the testing cohort. The H-score demonstrated superior predictive performance, with a c-statistic of 0.63 (95% CI: 0.61–0.65), outperforming both the HCT-CI (0.53; 95% CI: 0.51–0.55) and the TCI score (0.52; 95% CI: 0.50–0.54). Nonetheless, the modest c-statistic highlights the current limitations in achieving highly precise individualized risk prediction. Although the DRI demonstrated comparable discrimination for LFS to the H-score (0.68; 95% CI: 0.66–0.70), the H-score showed a broadly similar pattern, with slightly more gradual transitions between adjacent risk groups. (Supplementary Fig. S1).

### Secondary outcomes

Regarding the secondary objectives, the H-score demonstrated strong, independent discriminatory power for OS) GvHD-free and relapse-free survival (GFRS), REL incidence, and NRM, as detailed in Supplementary Tables S5 (training cohort) and S6 (testing cohort). In the multivariate model applied to the testing dataset adjusted for the H-score and other relevant covariates, the use of a 9/10 mismatched unrelated donor (compared to a matched sibling donor) and the use of a female donor for a male recipient were both associated with reduced OS (HR 1.27; 95% CI: 1.07–1.51 and HR 1.22; 95% CI: 1.06–1.40, respectively), as well as increased NRM (HR 1.61; 95% CI: 1.24–2.08 and HR 1.29; 95% CI: 1.05–1.58, respectively).

## Discussion

Despite the inherent complexity of the post-transplant course, physicians are tasked with making accurate predictions to effectively guide clinical decision-making [10]. Physicians strive to offer their patients the best possible chance of cure, while fully acknowledging that transplantation may not be curative and can lead to relapse or in serious morbidity or even mortality. However, clinical judgment in estimating the curative potential of transplantation, reflected by the probability of LFS, remains largely subjective and is often inaccurate or overly optimistic [11, 12]. In this study, we integrated prognostic information from established transplant-specific models into a single, holistic “H-score” specifically designed to predict LFS, the outcome that best reflects the curative intent of allogeneic transplantation. Given that acute leukemia remains the leading indication for allo-HCT and that transplant practices continue to evolve, we focused our analysis on patients with AML and ALL treated during the contemporary transplant era.

Transplantation is a multifactorial process in which pre-transplant factors, including patient characteristics, disease features, and transplantation procedures, have consistently been shown across numerous cohorts to independently influence post-transplant outcomes. Over the past two decades, various prognostic models have been developed to capture subsets of these variables and consolidate them into predictive scores. These tools have been widely adopted and validated for stratifying patients into distinct risk categories associated with specific transplant outcomes. In addition to age and KPS, which assess functional impairment, the HCT-CI model quantifies a patient’s comorbidity burden, serves as a predictor of NRM, and has been shown to correlate with reduced post-transplant survival at higher scores [2]. The DRI accounts for disease heterogeneity and pre-transplant disease status, classifying patients according to their risk of REL and OS [3, 6]. The TCI score refines classification of conditioning regimen intensity by capturing contemporary practices and predicting both NRM and REL, but due to the opposing effects of regimen intensity on these outcomes, it does not effectively stratify overall survival (OS) [4, 7]. The advantage of the TCI score over the traditional reduced-intensity conditioning (RIC) versus myeloablative conditioning (MAC) classification lies in its ability to capture reduced-toxicity myeloablative regimens, thereby more accurately reflecting the physician’s nuanced decision-making regarding the transplant approach. Since each of these models focuses on a specific subset of risk factors while overlooking other important variables that influence outcomes, their utility for personalized prediction of LFS remains limited. Shouval et al. compared eight prognostic scores within a single cohort of allogeneic transplant recipients and found that each score’s performance varied not only by the specific outcome assessed but also across different clinical contexts (e.g., RIC vs. MAC regimens). These findings highlight that, while such scores provide valuable information, they capture only a portion of the complexity shaping pre-transplant prognostic assessment [13]. In clinical practice, these tools complement each other in guiding decision-making around the transplantation procedure.

In this study, we integrated routinely assessed pre-transplant prognostic variables, including age, KPS, HCT-CI, DRI, and TCI, into a single, holistic H-score, and evaluated its association with LFS in a large, contemporary cohort of acute leukemia transplant recipients. While similar efforts to combine transplant-specific risk factors for outcome prediction have been reported, previous models have been limited by their reliance on relatively small, single-center cohorts from studies conducted prior to 2012. Moreover, none of the previously published models incorporated conditioning intensity, as done in our study. Specifically, the Pre-Transplantation Assessment of Mortality (PAM) score and the modified AML-specific EBMT score which was adapted from the original EBMT score developed two decades ago for chronic myeloid leukemia, include variables such as age, disease status, and donor type, but do not account for pre-transplant comorbidities [14–16]. Other models have combined the HCT-CI with components of the modified EBMT score or with disease-related variables, such as disease status at transplant or the DRI [17–20]. Using a machine learning model incorporating 10 variables, including RIC/MAC conditioning intensity, the EBMT developed a predictive score based on a large cohort of acute leukemia patients who underwent transplantation. However, this model was designed to predict day-100 mortality, was derived from transplants performed between 2000 and 2011, and notably did not account for pre-transplant comorbidities [21]. Differing from the above-mentioned approaches, the H-score was developed using data from a large cohort of contemporarily treated patients and integrates comprehensive prognostic information across all major domains, including patient status (age, KPS, HCT-CI), disease features (DRI), and procedural characteristics (TCI).

The current analysis, based on nearly 25,000 contemporary transplants, offers real-world, up-to-date prognostic estimates for allogeneic HCT in acute leukemia. The median 2-year OS of 64% and LFS of 56% are consistent with outcomes reported by other registries and single-center studies. Notably, the cumulative incidence of NRM at 15% reflects substantial progress in modern transplant practice, representing a significant improvement compared to the approximately 25% NRM observed in earlier transplant eras [22]. Using the H-score, patients were stratified into four risk groups, with 2-year LFS decreasing in a systematic, stepwise manner from 68.4% in the low-risk group to 58.6%, 48.2%, and 33.6% in the intermediate-, high-, and very high-risk groups, respectively. In a Cox regression analysis adjusted for additional known outcome-related factors, including donor type, stem cell source, CMV status, and female-to-male mismatch, the H-score emerged not only as a highly significant predictor of LFS (*p* < 0.0001), but also as the sole independent determinant, suggesting that it effectively consolidates key prognostic information and may serve as a standalone model for risk stratification. By contrast, the mono-parametric HCT-CI was unable to stratify survival in our cohort, likely reflecting physicians’ improved ability to individualize transplant strategies particularly for older or less fit patients, an observation consistent with findings from other studies [23]. The DRI demonstrated c-statistic performance comparable to the H-score in stratifying and predicting LFS, underscoring the pivotal role of disease status at the time of transplant as a key determinant of transplant failure. However, compared to the DRI, the H-score provided more balanced and clinically meaningful risk stratification, with differences in LFS between adjacent risk groups reaching up to 21%.

Key strengths of the H-score model include its development based on a large cohort of nearly 25,000 patients representative of contemporary practice, the integration of all major established pre-transplant prognostic scores, and its demonstrated validity and robustness for risk stratification across large training and testing datasets. Nonetheless, several limitations should be acknowledged. As with previous models, the H-score demonstrated limited discriminatory capacity (c-statistic 0.63), highlighting its suboptimal utility for individualized risk prediction. While this may suggest that the predictive utility of pre-transplant variables has reached its limits, more accurate personalized prognostication will likely emerge through the integration of biomarkers (e.g., somatic mutations, minimal residual disease), inclusion of GVHD prophylaxis (e.g., PTCY vs CNI-based), incorporation of less quantifiable factors (e.g., frailty, social vulnerability, cognitive impairment, sarcopenia), all of which may influence outcomes but were not included in the current model, along with improvements in data quality and the application of machine learning techniques to large-scale datasets [24–29]. However, it is important to recognize that, beyond fixed pre-transplant factors influencing LFS, outcomes are also shaped by numerous evolving post-transplant variables that cannot be fully anticipated at the time of transplantation. These include post-transplant maintenance strategies and relapse prophylaxis (e.g., donor lymphocyte infusions, targeted inhibitors), the quality of supportive care, caregiver support, and other social determinants of health, and unforeseen events such as infection outbreaks [30–32]. Therefore, the H-score should be interpreted as a pre-transplant baseline prognostic tool rather than a comprehensive model of all factors that influence post-transplant outcomes.

In clinical practice, the H-score’s greatest utility may lie in supporting physicians during informed consent discussions by providing patients and their loved ones with up-to-date, real-world estimates of their individual chances of cure. Additionally, the H-score may help identify patients who are least likely to benefit from allo-HCT, thereby guiding consideration of alternative therapeutic strategies or enrollment in clinical trials. As novel therapeutic approaches continue to evolve, the risk–benefit assessment for allogeneic transplantation becomes increasingly challenging. The transplant-specific H-score may offer a valuable baseline for contemporary transplant outcomes, serving as a reference point against which the efficacy of new treatment approaches can be compared.

## Supplementary information

Supplemental

## Data Availability

The data supporting this study are available from the European Society for Blood and Marrow Transplantation (EBMT) upon reasonable request. Requests should be directed to the EBMT Paris Study Unit (allain.ferhat@ebmt.org).
