# Supplementary material for: A holistic prognostic model for leukemia-free survival after allogeneic transplantation in acute leukemia
Source: Bone Marrow Transplant. 2026 Jul 2;61(9):1220–7. doi: 10.1038/s41409-026-02950-w (PMC13569450; doi:10.1038/s41409-026-02950-w)
Supplement: Supplementary file 1 [file 41409_2026_2950_MOESM1_ESM.pdf]

A Holistic Prognostic Model for Leukemia-Free Survival After Allogeneic Transplantation in Acute Leukemia

Supplemental Tables and Figures

Table S1: Detailed Characteristics of the Study Population

Variable	N	Overall, N = 24317	AML N = 19062	ALL N = 5255	p
**Transplant information**					
**Year of HCT**	24317				<0.001
Median		2019	2019	2019	
Range		2015, 2023	2015, 2023	2015, 2023	
**Time from diagnosis to HCT (mo)**	24309				<0.001
Median		5.7	5.4	6.7	
(IQR)		(4.2, 9.0)	(4.0, 8.4)	(5.2, 11.2)	
Range		0.0, 610.3	0.0, 610.3	0.4, 290.7	
Missing		8	8	0	
**Cell source**	24317				<0.001
PB		21976 (90%)	17405 (91%)	4571 (87%)	
BM		2341 (9.6%)	1657 (8.7%)	684 (13%)	
**Type of donors**	24317				<0.001
MSD		7353 (30%)	5528 (29%)	1825 (35%)	
Haplo		4459 (18%)	3521 (18%)	938 (18%)	
UD 9/10		2506 (10%)	1962 (10%)	544 (10%)	
UD 10/10		9999 (41%)	8051 (42%)	1948 (37%)	
**Transplant number**	24317				
First		24317 (100%)	19062 (100%)	5255 (100%)	
**Disease information**					
**Status at HCT**	24317				<0.001
CR1		17312 (71%)	13498 (71%)	3814 (73%)	
CR2+		3805 (16%)	2614 (14%)	1191 (23%)	
Active disease		3200 (13%)	2950 (15%)	250 (4.8%)	
**Cytogenetic AML**	19062				>0.99
Favorable		1295 (6.8%)	1295 (6.8%)	0 (NA%)	
Intermediate		13461 (71%)	13461 (71%)	0 (NA%)	
Adverse		4306 (23%)	4306 (23%)	0 (NA%)	
Missing		5255	0	5255	

**Variable**	**N**	**Overall, N = 24317**	**AML N = 19062**	**ALL N = 5255**	**p**
**Patient and donor information**					
**Patient Age at HCT**	24317				<0.001
Median (years)		53.2	55.9	40.6	
IQR		(39.9, 62.3)	(44.5, 63.7)	(28.0, 53.0)	
Range		18.0, 75.0	18.0, 75.0	18.0, 75.0	
**Patient (P) Sex**	24285				<0.001
Female		10872 (45%)	8731 (46%)	2141 (41%)	
Male		13413 (55%)	10305 (54%)	3108 (59%)	
Missing		32	26	6	
**Sex of the Donor (D)**	24202				0.26
Female		8509 (35%)	6641 (35%)	1868 (36%)	
Male		15693 (65%)	12346 (65%)	3347 (64%)	
Missing		115	75	40	
**Female Donor to male**	24232				<0.001
No		20074 (83%)	15864 (83%)	4210 (80%)	
Yes		4158 (17%)	3135 (17%)	1023 (20%)	
Missing		85	63	22	
**Patient CMV**	24111				<0.001
Negative		7531 (31%)	5789 (31%)	1742 (33%)	
Positive		16580 (69%)	13120 (69%)	3460 (67%)	
Missing		206	153	53	
**Donor CMV**	24000				0.037
Negative		10747 (45%)	8494 (45%)	2253 (44%)	
Positive		13253 (55%)	10327 (55%)	2926 (56%)	
Missing		317	241	76	
**CMV Donor to Patient**	23862				<0.001
Neg to Neg		5497 (23%)	4228 (23%)	1269 (25%)	
Neg to Pos		5176 (22%)	4209 (22%)	967 (19%)	
Pos to Neg		1973 (8.3%)	1512 (8.1%)	461 (9.0%)	
Pos to Pos		11216 (47%)	8769 (47%)	2447 (48%)	
Missing		455	344	111	
**CMV Donor /Patient**	23862				0.002
Neg / Neg		5497 (23%)	4228 (23%)	1269 (25%)	
Other		18365 (77%)	14490 (77%)	3875 (75%)	
Missing		455	344	111	
**Conditioning Information**					
RIC or MAC regimen	24206				<0.001
RIC		9389 (39%)	8438 (45%)	951 (18%)	

Variable	N	Overall, N = 24317	AML N = 19062	ALL N = 5255	p
MAC-TBI		4452 (18%)	1250 (6.6%)	3202 (61%)	
MAC-Chemo		10365 (43%)	9269 (49%)	1096 (21%)	
Missing		111	105	6	
**Conditioning regimen**	24317				<0.001
TBI-based		6032 (25%)	2579 (14%)	3453 (66%)	
TBF-based		3903 (16%)	3271 (17%)	632 (12%)	
TreoFlu-based		2482 (10%)	2386 (13%)	96 (1.8%)	
BuFlu-based		7144 (29%)	6689 (35%)	455 (8.7%)	
BuCy-based		1841 (7.6%)	1675 (8.8%)	166 (3.2%)	
FluMel-based		2388 (9.8%)	2014 (11%)	374 (7.1%)	
Other		527 (2.2%)	448 (2.4%)	79 (1.5%)	
**GvHD Prevention Information**					
**Main Prevention**	24215				<0.001
ATG		11575 (48%)	9524 (50%)	2051 (39%)	
PTCY		5202 (21%)	4055 (21%)	1147 (22%)	
PTCY+ATG		600 (2.5%)	481 (2.5%)	119 (2.3%)	
Other		6838 (28%)	4921 (26%)	1917 (37%)	
Missing		102	81	21	
**Associated Prevention**	24317				
CSA-based		2624 (11%)	2043 (11%)	581 (11%)	
MTX-based		14 (<0.1%)	11 (<0.1%)	3 (<0.1%)	
MMF-based		180 (0.7%)	148 (0.8%)	32 (0.6%)	
MMF+CSA-based		8066 (33%)	6834 (36%)	1232 (23%)	
MMF+CSA+TACRO-bas		123 (0.5%)	99 (0.5%)	24 (0.5%)	
MMF+SIRO-based		350 (1.4%)	299 (1.6%)	51 (1.0%)	
MMF+TACRO-based		2368 (9.7%)	1902 (10.0%)	466 (8.9%)	
MTX+CSA-based		8711 (36%)	6314 (33%)	2397 (46%)	
MTX+CSA+TACRO-bas		72 (0.3%)	42 (0.2%)	30 (0.6%)	
MTX+MMF-based		23 (<0.1%)	17 (<0.1%)	6 (0.1%)	
MTX+TACRO-based		503 (2.1%)	354 (1.9%)	149 (2.8%)	
TACRO-based		555 (2.3%)	428 (2.2%)	127 (2.4%)	
TACRO+SIRO-based		146 (0.6%)	121 (0.6%)	25 (0.5%)	
MTX+SIRO-based		2 (<0.1%)	2 (<0.1%)	0 (0%)	
Other		580 (2.4%)	448 (2.4%)	132 (2.5%)	

Table S2: Estimation of outcomes

All patients (N= 24,317)					
	**OS (2y)**	**LFS (2y)**	**GRFS (2y)**	**RI (2y)**	**NRM (2y)**
**Overall**	64 (63.3 - 64.6)	56 (55.3 - 56.7)	43.2 (42.5 - 43.9)	29 (28.4 - 29.7)	15 (14.5 - 15.5)
**AML**	63.1 (62.4 - 63.9)	55.8 (55 - 56.6)	43.2 (42.4 - 44)	29.4 (28.7 - 30.2)	14.8 (14.3 - 15.4)
**ALL**	67 (65.5 - 68.4)	56.8 (55.2 - 58.3)	43.2 (41.6 - 44.7)	27.6 (26.2 - 29)	15.6 (14.6 - 16.7)
**HCT-CI**					
**0**	67.4 (66.4 - 68.3)	58.5 (57.5 - 59.4)	45.7 (44.8 - 46.7)	28.7 (27.8 - 29.6)	12.9 (12.2 - 13.5)
**1-2**	62.9 (61.5 - 64.3)	55.9 (54.4 - 57.4)	43.2 (41.7 - 44.7)	27.9 (26.6 - 29.2)	16.2 (15.1 - 17.3)
**>=3**	57.7 (56.3 - 59.1)	50.7 (49.3 - 52.2)	37.7 (36.3 - 39.1)	30.9 (29.5 - 32.2)	18.4 (17.3 - 19.5)
**DRI**					
**Low**	76.3 (73.4 - 79)	66.9 (63.7 - 69.9)	52.8 (49.5 - 56.1)	21.6 (18.9 - 24.4)	11.5 (9.6 - 13.7)
**Int**	70.6 (69.8 - 71.4)	62.6 (61.7 - 63.5)	48.9 (48 - 49.8)	23.3 (22.6 - 24.1)	14.1 (13.5 - 14.7)
**High**	52.5 (51.2 - 53.9)	44.3 (42.9 - 45.7)	33.1 (31.8 - 34.4)	39 (37.6 - 40.3)	16.7 (15.8 - 17.7)
**Very high**	30.2 (27.3 - 33.1)	24.4 (21.7 - 27.3)	16.4 (14.1 - 18.8)	55.2 (52 - 58.3)	20.3 (17.9 - 22.9)
**TCI**					
**1-2**	60.5 (59.2 - 61.9)	53.2 (51.8 - 54.5)	41.7 (40.3 - 43)	32.1 (30.8 - 33.4)	14.7 (13.8 - 15.7)
**2.5-3.5**	62.8 (61.8 - 63.8)	55.6 (54.6 - 56.6)	43.6 (42.6 - 44.7)	27.8 (26.9 - 28.7)	16.6 (15.9 - 17.4)
**4-6**	69.7 (68.4 - 71)	59.7 (58.3 - 61.1)	43.9 (42.5 - 45.3)	28 (26.7 - 29.3)	12.3 (11.4 - 13.2)
**Donor**					
**MSD**	66.5 (65.3 - 67.8)	57 (55.7 - 58.3)	41.7 (40.4 - 43)	31.9 (30.7 - 33.1)	11.1 (10.3 - 11.9)
**Haplo**	60.1 (58.5 - 61.7)	54.8 (53.1 - 56.5)	43.4 (41.7 - 45)	25.4 (24 - 26.9)	19.8 (18.5 - 21.1)
**UD 10/10**	64.9 (63.8 - 65.9)	56.6 (55.4 - 57.7)	45 (43.9 - 46.1)	28.8 (27.8 - 29.8)	14.7 (13.9 - 15.4)
**UD 9/10**	59.6 (57.4 - 61.7)	52.8 (50.5 - 54.9)	39.8 (37.6 - 41.9)	28 (26 - 30)	19.2 (17.6 - 21)

Table S3: Multivariable analysis of LFS for the training dataset (N=19,029)

Outcome	Variable	Reference	Level	HR (95%CI)	P-value	Signif
**LFS**	HCT-CI	0	1-2	1.07 (1.01-1.13)	0.029	*
**LFS**	HCT-CI	0	>=3	1.23 (1.16-1.3)	<0.001	***
**LFS**	DRI	Low	Int	1.11 (0.98-1.26)	0.088	
**LFS**	DRI	Low	High	1.96 (1.73-2.22)	<0.001	***
**LFS**	DRI	Low	Very high	3.74 (3.22-4.34)	<0.001	***
**LFS**	Class of TCI	4-6	2.5-3.5	1.04 (0.98-1.11)	0.197	
**LFS**	Class of TCI	4-6	1-2	1.14 (1.06-1.23)	0.001	**
**LFS**	Age of the Patient at HCT	<=55	>55	1.15 (1.09-1.22)	<0.001	***
**LFS**	Karnofsky score	>= 90	< 90	1.2 (1.14-1.27)	<0.001	***
**LFS**	Type of AL	AML	ALL	1.06 (1-1.12)	0.057	
**LFS**	Type of donors	MSD	Haplo	1.02 (0.95-1.09)	0.598	
**LFS**	Type of donors	MSD	UD 9/10	1.1 (1.01-1.19)	0.023	*
**LFS**	Type of donors	MSD	UD 10/10	1.01 (0.95-1.07)	0.846	
**LFS**	Cell source	BM	PB	1.1 (1.01-1.19)	0.024	*
**LFS**	Female donor to male patient	No	Yes	1.02 (0.96-1.08)	0.618	
**LFS**	CMV Donor to patient	Neg to Neg	Other	1.09 (1.03-1.15)	0.005	**

Table S4: Outcomes by H-score

Training data set (N= 19,029)					
**H-score**	**OS (2y)**	**LFS (2y)**	**GRFS (2y)**	**RI (2y)**	**NRM (2y)**
**Overall**	64.4 (63.6 - 65.2)	56.4 (55.6 - 57.2)	43.9 (43.1 - 44.6)	29 (28.3 - 29.7)	14.6 (14 - 15.1)
**0-1**	79 (77 - 80.9)	69.1 (66.8 - 71.3)	51.4 (48.9 - 53.8)	21.8 (19.8 - 23.9)	9.1 (7.8 - 10.6)
**2-3**	70.1 (69 - 71.2)	61 (59.8 - 62.1)	48.2 (47.1 - 49.4)	26.7 (25.7 - 27.8)	12.3 (11.6 - 13.1)
**4-5**	56 (54.7 - 57.3)	49.7 (48.3 - 51)	38.4 (37.1 - 39.6)	31.9 (30.7 - 33.1)	18.4 (17.5 - 19.5)
**6-7**	31.6 (27.7 - 35.6)	27.7 (23.8 - 31.6)	20.4 (17.1 - 24)	51.3 (47 - 55.4)	21.1 (17.8 - 24.5)
**Testing data set (N= 4,760)**					
**H-score**	**OS (2y)**	**LFS (2y)**	**GRFS (2y)**	**RI (2y)**	**NRM (2y)**
**Overall**	62.7 (61.1 - 64.3)	54.8 (53.1 - 56.4)	40.8 (39.2 - 42.4)	28.7 (27.2 - 30.2)	16.5 (15.3 - 17.7)
**0-1**	77.3 (71.6 - 82)	66.2 (59.9 - 71.8)	50.4 (44.1 - 56.3)	20.4 (15.5 - 25.7)	13.4 (9.6 - 17.8)
**2-3**	72.3 (69.7 - 74.6)	62.2 (59.5 - 64.8)	47.5 (44.7 - 50.1)	27.6 (25.2 - 30.1)	10.2 (8.7 - 11.8)
**4-5**	58.9 (56.4 - 61.3)	52.3 (49.8 - 54.8)	38.6 (36.2 - 41)	28.6 (26.3 - 30.8)	19.2 (17.3 - 21.1)
**6-7**	36.2 (31.5 - 40.9)	31.8 (27.1 - 36.5)	21.2 (17.3 - 25.4)	38.4 (33.6 - 43.2)	29.8 (25.5 - 34.3)

Figure S1. Leukemia free survival (LFS) according to H-score, DRI, HCT-CI and TCI in the training (A) and testing population (B)

Fig. S1A

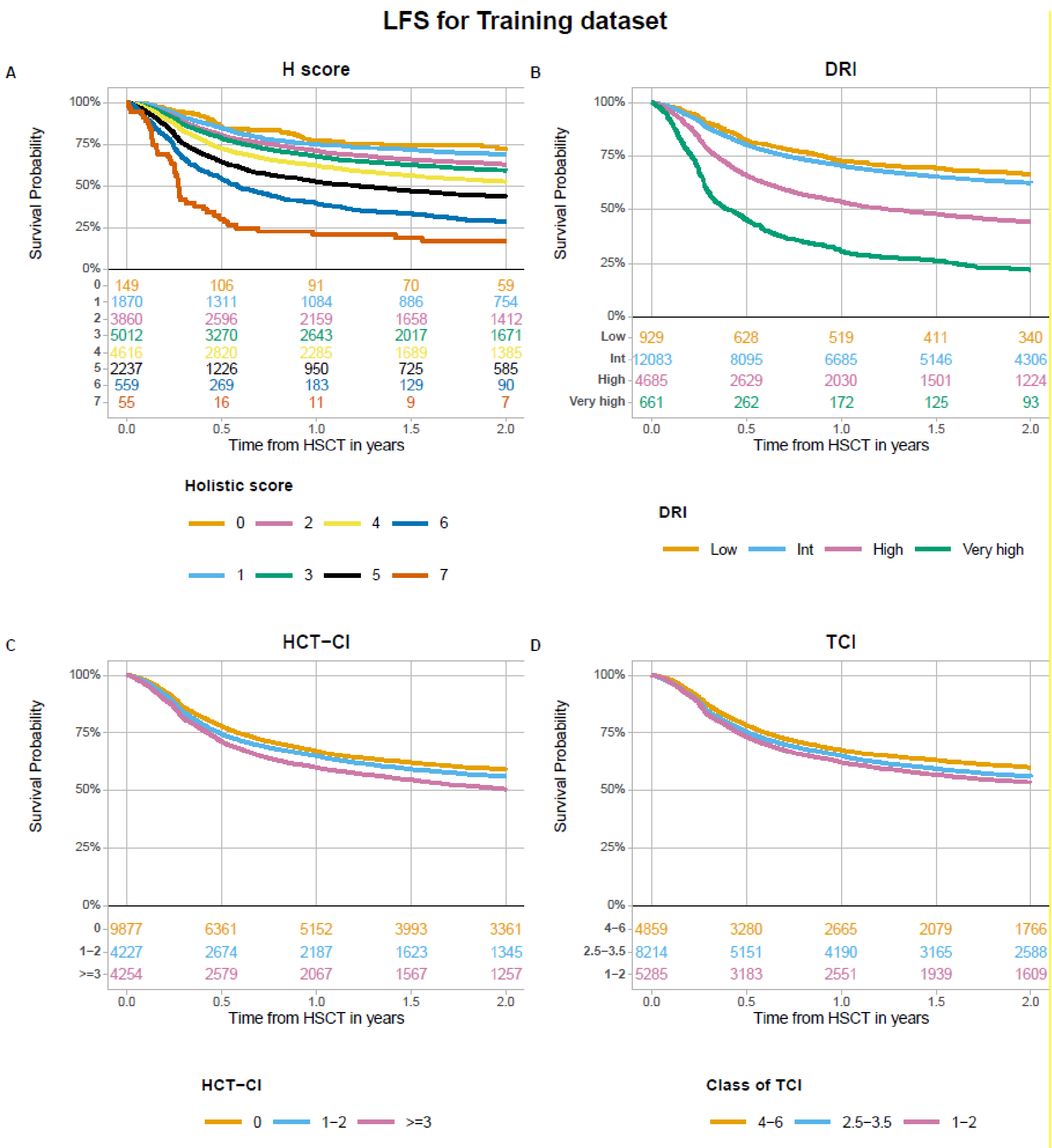

Fig. S1B

LFS for Testing dataset

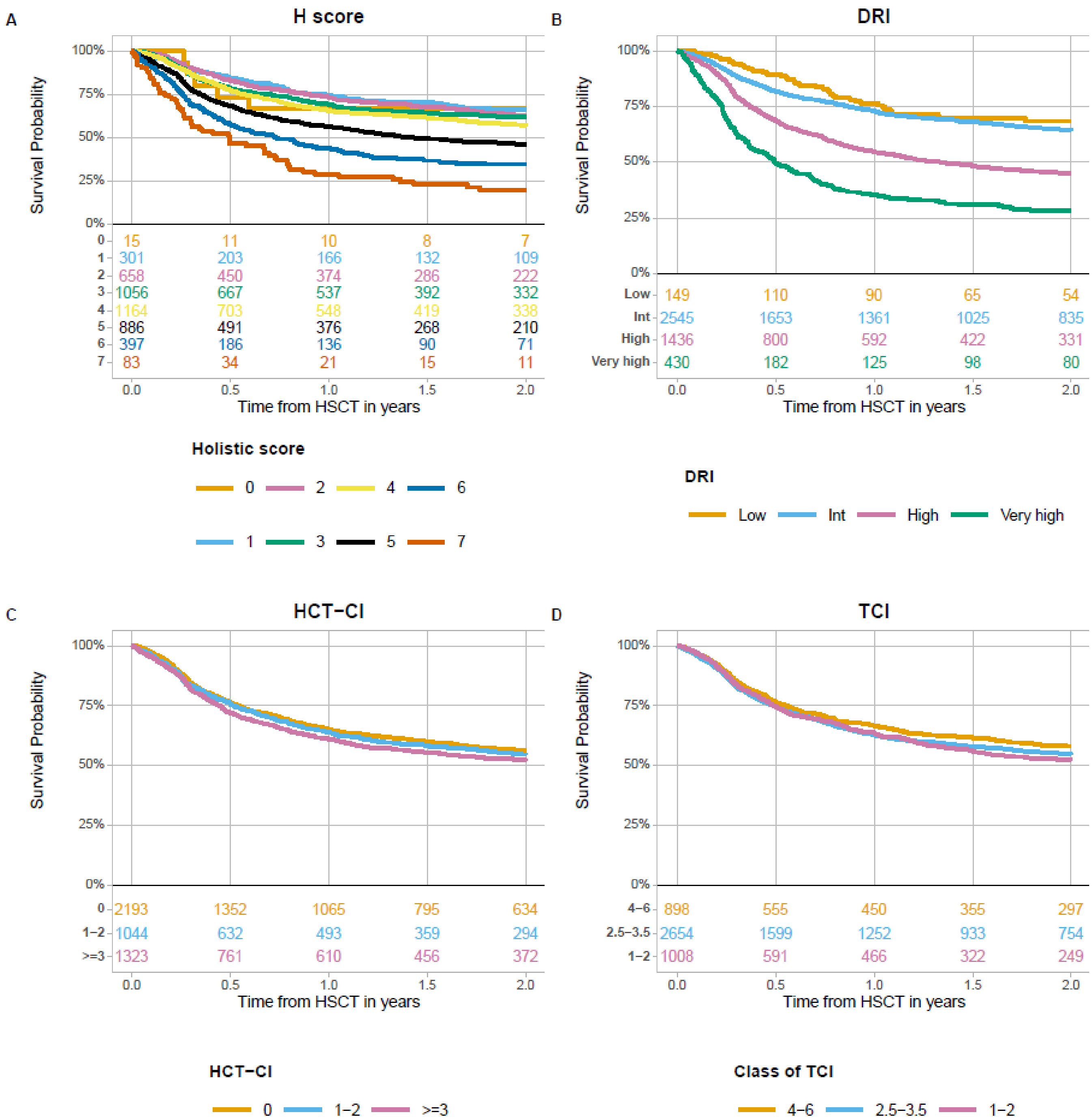

Table S5: Multivariable analysis of outcomes for the training dataset (N=19,029)

	OS		LFS		GRFS		RI		NRM	
**Variable**	**HR (95%CI)**	**P-value**	**HR (95%CI)**	**P-value**	**HR (95%CI)**	**P-value**	**HR (95%CI)**	**P-value**	**HR (95%CI)**	**P-value**
**Holistic score group: 0-1 VS 2-3**	1.45 (1.31-1.6)	<0.001	1.36 (1.25-1.49)	<0.001	1.12 (1.04-1.21)	0.002	1.37 (1.23-1.52)	<0.001	1.33 (1.13-1.56)	<0.001
**Holistic score group: 0-1 VS 4-5**	2.42 (2.19-2.68)	<0.001	2.01 (1.84-2.2)	<0.001	1.5 (1.39-1.61)	<0.001	1.87 (1.68-2.09)	<0.001	2.26 (1.93-2.66)	<0.001
**Holistic score group: 0-1 VS 6-7**	4.94 (4.31-5.66)	<0.001	3.87 (3.4-4.39)	<0.001	2.52 (2.25-2.82)	<0.001	4.12 (3.53-4.8)	<0.001	3.33 (2.63-4.2)	<0.001
**Type of donors: MSD VS Haplo**	1.21 (1.12-1.3)	<0.001	1.02 (0.95-1.09)	0.545	1.01 (0.95-1.07)	0.863	0.75 (0.69-0.82)	<0.001	1.85 (1.64-2.08)	<0.001
**Type of donors: MSD VS UD 10/10**	1.06 (1-1.13)	0.065	0.98 (0.93-1.04)	0.566	0.92 (0.88-0.97)	0.002	0.85 (0.79-0.91)	<0.001	1.41 (1.27-1.57)	<0.001
**Type of donors: MSD VS UD 9/10**	1.28 (1.17-1.4)	<0.001	1.1 (1.01-1.19)	0.026	1.08 (1.01-1.17)	0.027	0.84 (0.76-0.93)	0.001	1.91 (1.66-2.19)	<0.001
**Cell source: BM VS PB**	1.16 (1.07-1.27)	0.001	1.08 (1-1.18)	0.047	1.11 (1.04-1.2)	0.002	1.06 (0.96-1.17)	0.216	1.14 (0.99-1.3)	0.066
**Female donor to male patient: No VS Yes**	1.07 (1-1.14)	0.04	1.02 (0.96-1.08)	0.623	1.06 (1.01-1.12)	0.026	0.93 (0.86-1)	0.044	1.21 (1.1-1.34)	<0.001
**CMV Donor to patient: Neg to Neg VS Other**	1.1 (1.03-1.17)	0.005	1.07 (1.01-1.14)	0.023	1.03 (0.98-1.09)	0.229	1.04 (0.97-1.11)	0.3	1.14 (1.03-1.27)	0.013

Table S4: Multivariable analysis of outcomes for the testing dataset (N=4,760)

	OS		LFS		GRFS		RI		NRM	
**Variable**	**HR (95%CI)**	**P-value**	**HR (95%CI)**	**P-value**	**HR (95%CI)**	**P-value**	**HR (95%CI)**	**P-value**	**HR (95%CI)**	**P-value**
**Holistic score group: 0-1 VS 2-3**	1.25 (0.97-1.6)	0.079	1.24 (1-1.55)	0.054	1.1 (0.92-1.32)	0.297	1.5 (1.13-2)	0.005	0.84 (0.59-1.19)	0.321
**Holistic score group: 0-1 VS 4-5**	2.23 (1.74-2.85)	<0.001	1.91 (1.53-2.38)	<0.001	1.49 (1.25-1.78)	<0.001	1.94 (1.46-2.58)	<0.001	1.75 (1.24-2.46)	0.001
**Holistic score group: 0-1 VS 6-7**	4.77 (3.66-6.22)	<0.001	3.72 (2.92-4.75)	<0.001	2.54 (2.08-3.11)	<0.001	3.62 (2.64-4.97)	<0.001	3.41 (2.35-4.95)	<0.001
**Type of donors: MSD VS Haplo**	1.1 (0.91-1.32)	0.318	1.02 (0.86-1.21)	0.85	0.87 (0.75-1.01)	0.07	0.83 (0.66-1.03)	0.096	1.46 (1.11-1.92)	0.007
**Type of donors: MSD VS UD 10/10**	1 (0.88-1.13)	0.973	0.98 (0.87-1.1)	0.753	0.81 (0.73-0.89)	<0.001	0.91 (0.79-1.05)	0.189	1.12 (0.92-1.37)	0.261
**Type of donors: MSD VS UD 9/10**	1.27 (1.07-1.51)	0.006	1.2 (1.02-1.41)	0.03	1.08 (0.94-1.24)	0.25	1 (0.82-1.24)	0.97	1.61 (1.24-2.08)	<0.001
**Cell source: BM VS PB**	0.85 (0.66-1.09)	0.208	0.76 (0.6-0.96)	0.021	0.9 (0.73-1.1)	0.302	0.7 (0.53-0.93)	0.015	0.88 (0.6-1.3)	0.528
**Female donor to male patient: No VS Yes**	1.22 (1.06-1.4)	0.005	1.13 (0.99-1.29)	0.063	1.08 (0.97-1.21)	0.163	1.04 (0.88-1.24)	0.617	1.29 (1.05-1.58)	0.016
**CMV Donor to patient: Neg to Neg VS Other**	0.99 (0.89-1.1)	0.802	1.01 (0.91-1.11)	0.886	0.99 (0.91-1.08)	0.882	0.99 (0.88-1.13)	0.908	1.03 (0.88-1.22)	0.687
